# Distinct Biochemical Activities of Eyes absent During *Drosophila* Eye Development

**DOI:** 10.1038/srep23228

**Published:** 2016-03-16

**Authors:** Meng Jin, Graeme Mardon

**Affiliations:** 1 Department of Pathology and Immunology, Baylor College of Medicine, Houston, TX 77030, USA; 2 Program in Developmental Biology, Baylor College of Medicine, Houston, TX 77030, USA; 3 Department of Molecular and Human Genetics, Baylor College of Medicine, Houston, TX 77030, USA; 4 Department of Neuroscience, Baylor College of Medicine, Houston, TX 77030, USA; 5 Department of Ophthalmology, Baylor College of Medicine, Houston, TX 77030, USA; 6 Program in Cell and Molecular Biology, Baylor College of Medicine, One Baylor Plaza, Houston, TX 77030, USA

## Abstract

Eyes absent (Eya) is a highly conserved transcriptional coactivator and protein phosphatase that plays vital roles in multiple developmental processes from *Drosophila* to humans. Eya proteins contain a PST (Proline-Serine-Threonine)-rich transactivation domain, a threonine phosphatase motif (TPM), and a tyrosine protein phosphatase domain. Using a genomic rescue system, we find that the PST domain is essential for Eya activity and Dac expression, and the TPM is required for full Eya function. We also find that the threonine phosphatase activity plays only a minor role during *Drosophila* eye development and the primary function of the PST and TPM domains is transactivation that can be largely substituted by the heterologous activation domain VP16. Along with our previous results that the tyrosine phosphatase activity of Eya is dispensable for normal Eya function in eye formation, we demonstrate that a primary function of Eya during *Drosophila* eye development is as a transcriptional coactivator. Moreover, the PST/TPM and the threonine phosphatase activity are not required for *in vitro* interaction between retinal determination factors. Finally, this work is the first report of an Eya-Ey physical interaction. These findings are particularly important because they highlight the need for an *in vivo* approach that accurately dissects protein function.

*Drosophila* eye development depends on a network of retinal determination (RD) genes, which encode conserved nuclear proteins that play critical roles in *Drosophila* eye development^1^. The core RD genes include *twin of eyeless* (*toy*)^2^, *eyeless* (*ey*)^3^, *eyes absent* (*eya*)^4^*, sine oculis* (*so*)^5^, and *dachshund* (*dac*)^6^. These genes are involved in interconnected feedback loops and their protein products are necessary and sufficient for inducing retinal fate. *ey* and *toy*, which encode paired-type homeobox genes, lie atop the genetic hierarchy controlling eye development. Ey activates the expression of *eya* and *so*, which in turn induce *dac*^7^. Eya and So expression in the eye begins during the second instar larval stage and is highest near the posterior margin. After morphogenetic furrow (MF) initiation, Eya and So are co-expressed within and posterior to the MF, as well as in a zone immediately anterior to it^4^,^5^. Once established, So maintains its own expression, as well as that of *eya*, *dac*, and *ey*. So associates with Eya and it is thought that Eya act as a transcriptional coactivator upon recruitment by So, since Eya has no recognized DNA binding activity, but possesses a transactivation domain^8^,^9^,^10^,^11^. Eya can also physically interact with Dac to regulate target genes during eye development^7^,^11^,^12^,^13^,^14^,^15^. A similar interaction has been reported between their mouse counterparts EYA2 and DACH2^16^.

As a key member of the RD gene network, Eya acts as a transcriptional coactivator and also contains both tyrosine and threonine phosphatase activities^17^,^18^,^19^. Eya regulates multiple developmental processes throughout the metazoans^15^. In the *Drosophila* eye, loss of *eya* function blocks MF initiation, causes massive apoptosis in eye discs, and the complete failure of eye development. This cell death phenotype resembles those seen in the ear and kidney primordia of *Eya1* mutant mouse embryos^20^. In contrast to these loss-of-function phenotypes, ectopic overexpression of *eya* in other imaginal discs is sufficient to cause the formation of ectopic eyes^21^.

*Drosophila* Eya contains a highly conserved C-terminal domain called the Eya Domain (ED) and a moderately conserved threonine phosphatase motif (TPM) embedded in a proline-serine-threonine-rich (PST) domain. Throughout the remainder of this paper, “PST/TPM”, “PST”, and “TPM” represent the PST domain with the TPM, the PST domain alone, and the TPM alone, respectively. Eya and So bind to each other through the ED of Eya and the Six domain of So^8^,^11^ to form a transcriptional activator complex. In addition, a series of *Drosophila* S2 cell-based transcriptional activation assays defined the PST/TPM domain as essential for Eya/So-mediated transactivation of a reporter. *UAS-eya* transgenes that lack both the PST-rich region and the TPM have drastically reduced ectopic eye-inducing capacity, with induction efficiency dropping from 98% to 1.5%^10^.

In addition to regulating transcription, Eya has predicted tyrosine and threonine phosphatase activities in the ED and TPM, respectively^17^,^18^,^19^,^22^,^23^,^24^. In *Drosophila*, tyrosine phosphatase-dead mutations lead to strongly reduced activities in ectopic eye induction and *in vivo* genetic rescue using the *GAL4-UAS* system^18^,^19^,^24^. In contrast to these studies, our previous findings revealed that *eya* genomic rescue (GR) constructs carrying mutations in two key tyrosine phosphatase active-site residues fully restore viability as well as eye formation and function in an *eya* null mutant background^25^. In mouse and *Drosophila*, the threonine phosphatase activity has been suggested to play an important role in the innate immune system^17^ and a recent study using the *GAL4-UAS* system reported that Eya threonine phosphatase activity is not required for normal *Drosophila* eye development^24^.

Although previous cell culture and *in vivo GAL4-UAS* based expression studies have suggested specific functions for conserved Eya domains, we have shown that such assays may not always be reliable. In particular, we have developed a genomic rescue (GR) system that provides an accurate method for assessing the functional significance of individual protein domains *in vivo*^25^,^26^. In this study, we have used the GR strategy to conduct functional studies of Eya domains during *Drosophila* eye development. Interestingly, we found that a major function of Eya is transcriptional coactivation, while the threonine phosphates activity plays only a minor role during *Drosophila* development.

## Results

### The threonine phosphatase activity of Eya plays only a minor role in normal *Drosophila* development

**Figure 1:**
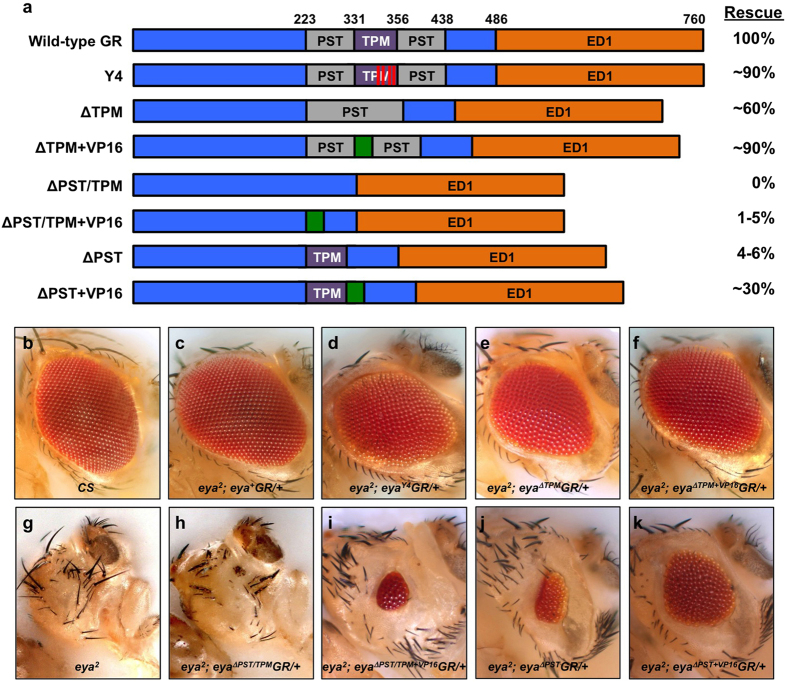
e*ya* genomic rescue constructs rigorously define Eya functional domains. (**a**) Schematic view of the *eya* genomic rescue (GR) constructs assayed in this study. The percent rescue indicated is for animals with one copy of each GR construct in an *eya*^2^ homozygous mutant background. At least 100 adult eyes were scored for each GR and the penetrance of the transgene-induced phenotypes for all GRs are 100%, with minor variation in expressivity. Each construct is inserted at the same genomic docking site such that all constructs are directly comparable. (**b–k**) Representative images of adult eyes for each genotype tested are shown. PST: grey box, TPM: purple box, Y4: red vertical lines, VP16: green box, ED1: orange box.

**Figure 2:**
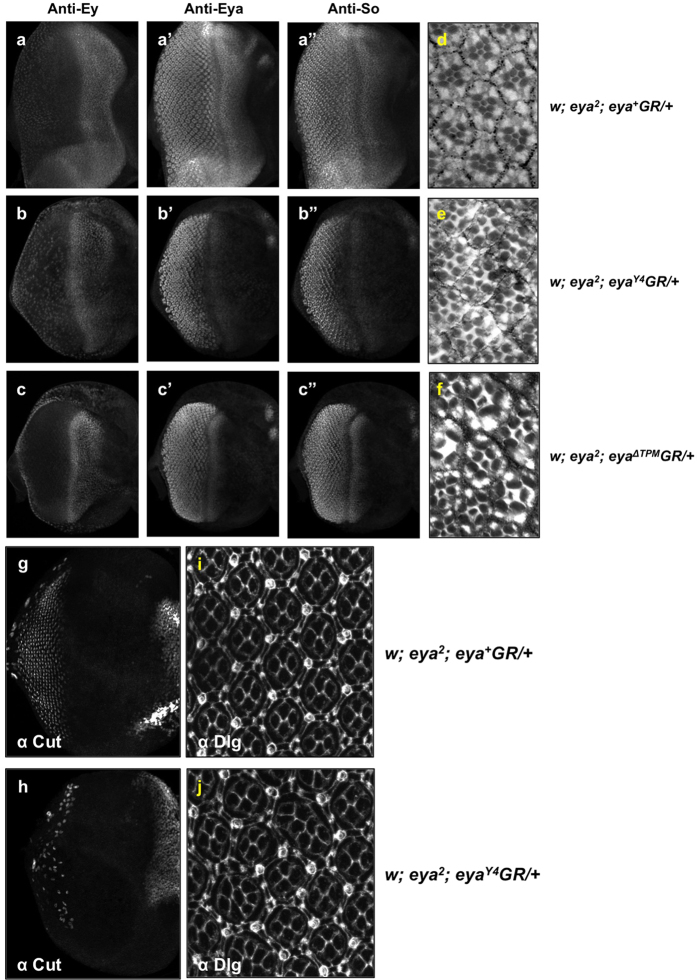
Threonine phosphatase activity is required for normal anterior expression of *eya* and cone cell development. Expression of Ey (**a–c**), Eya (**a’–c’**), and So (**a”–c”**) proteins are shown in *eya*^+^*GR*, *eya*^*Y4*^*GR*, and *eya*^Δ*TPM*^*GR* rescued animals. (**d–f**) Adult plastic sections in flies rescued with one copy of *eya*^+^*GR*, *eya*^*Y4*^*GR* and *eya*^Δ*TPM*^*GR*, respectively. (**g,h**) Third instar eye discs by Cut staining. (**I,j**) Eye discs prepared from 48 hrs after puparium formation and stained with Dlg.

**Figure 3:**
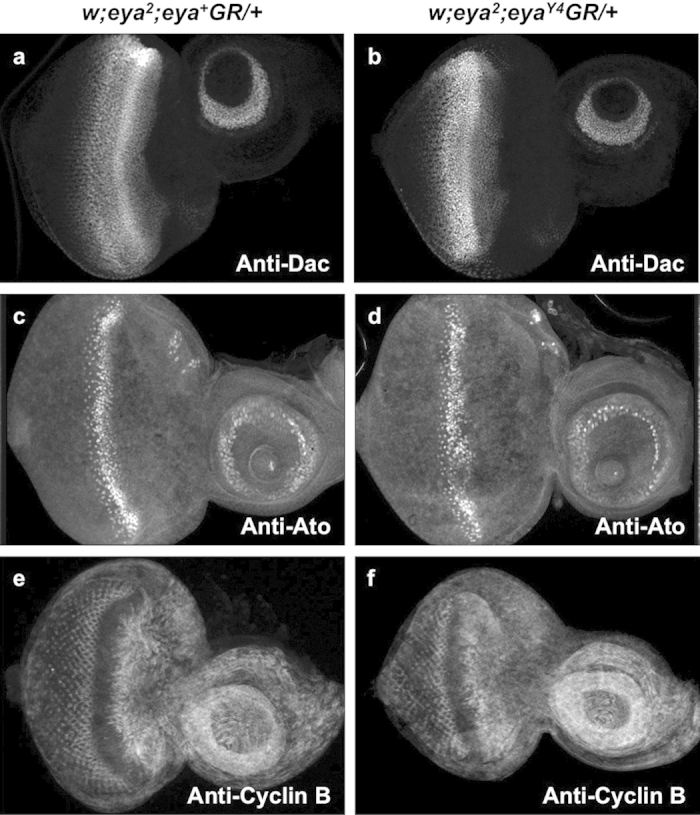
Loss of threonine phosphatase activity does not strongly affect Dac, Ato, or Cyclin B expression. Dac (**a**,**b**), Ato (**c**,**d**), and the cell cycle marker Cyclin B (**e**,**f**) expression in animals rescued with a single copy of *eya*^+^*GR* or *eya*^*Y4*^*GR* are shown. Expression patterns and levels of Eya, Ato and Cyclin B are similar in both genotypes although minor disruption of Ato is observed.

To study *eya* function *in vivo*, we introduced a series of *eya* genomic rescue constructs (*eyaGR*) via site-specific transgenesis^27^,^28^ to investigate the transcriptional activation and threonine phosphatase activity of Eya. A wild-type *eya* genomic rescue construct (*eya*^+^*GR*) is known to fully rescue viability and eye formation in an *eya* null mutant background, therefore serving as a positive control throughout our studies^25^,^26^,^29^. The *eya*^*Y4*^*GR* construct has tyrosine-to-alanine substitutions for four key tyrosine residues known to be required for threonine phosphatase activity^17^,^24^ (Fig. 1a). The *eya*^Δ*TPM*^*GR* construct has the entire TPM deleted but leaves the PST domain intact. Surprisingly, a single copy of each construct is able to substantially rescue *eya*^2^ or *eya*^*cliIID*^ mutant phenotypes, restoring viability and rescuing eye size to ~90% (*Y4*) or ~60% (Δ*TPM*) of wild-type, albeit with some mild disorganization (Fig. 1d,e and Fig. S1). While there appears to be a largely normal complement and arrangement of rhabdomeres in ommatidia of *eya*^2^*; eya*^*Y4*^*GR/*+ flies (Fig. 2e), eye discs from late third instar larvae (Fig. 2h) and 24 hrs after puparium formation (Fig. 2j) show defects in the number of cone cells and/or ommatidial fusion. Larval eye discs from *eya*^*Y4*^*GR* and *eya*^Δ*TPM*^*GR* rescued animals are smaller and show a reduction in Eya and So staining anterior to and within the MF while expression levels are normal posteriorly (Fig. 2b’”), suggesting that the threonine phosphatase activity does play a role during *Drosophila* eye development but this role is relatively minor as the *eya*^*Y4*^*GR* construct can restore up to 90% of the eye size. The expression of the core RD genes Dachshund (Dac) and Eyeless (Ey) appear similar in eye discs of positive control and *eya*^*Y4*^*GR*-rescued larvae (Figs 2a–c and 3a,b). In addition, we found no difference in photoreceptor axon projections between wild-type and *eya*^2^*; eya*^*Y4*^*GR/*+ flies (Fig. S2c), which show a regular pattern of projections in the lamina of the optic lobe.

*eya* plays an important role in the developmental events associated with morphogenetic furrow movement. Specifically, clonal analysis has shown that *eya* is required for the initiation and propagation of the MF and for regulation of the cell cycle^4^,^8^,^30^,^31^. Since loss of threonine phosphatase activity leads to a reduction of Eya expression anterior to and within the MF, we analyzed the effects of *eya*^*Y4*^ on both G1-arrest and induction of the proneural gene *atonal* (*ato*). We used the cell cycle marker Cyclin B to monitor G1 arrest. Normally, Cyclin B is exclusively expressed in cells in the G2 and M phases^32^. Immunohistochemistry shows *eya*^*Y4*^*GR* rescued animals have largely normal Cyclin B and Ato expression patterns (Fig. 3c–f), implying that the threonine phosphatase-inactive mutations do not adversely affect G1 arrest and initiation of retinal differentiation. This is not surprising since the loss of retinal cells in flies rescued with a single copy of *eya*^*Y4*^*GR* is relatively mild; therefore, strong alterations in the expression of markers of cell cycle progression or photoreceptor differentiation are not expected.

### The Eya threonine phosphatase-inactive mutation does not abolish interaction of Eya with Ey, So, or Dac

**Figure 4:**
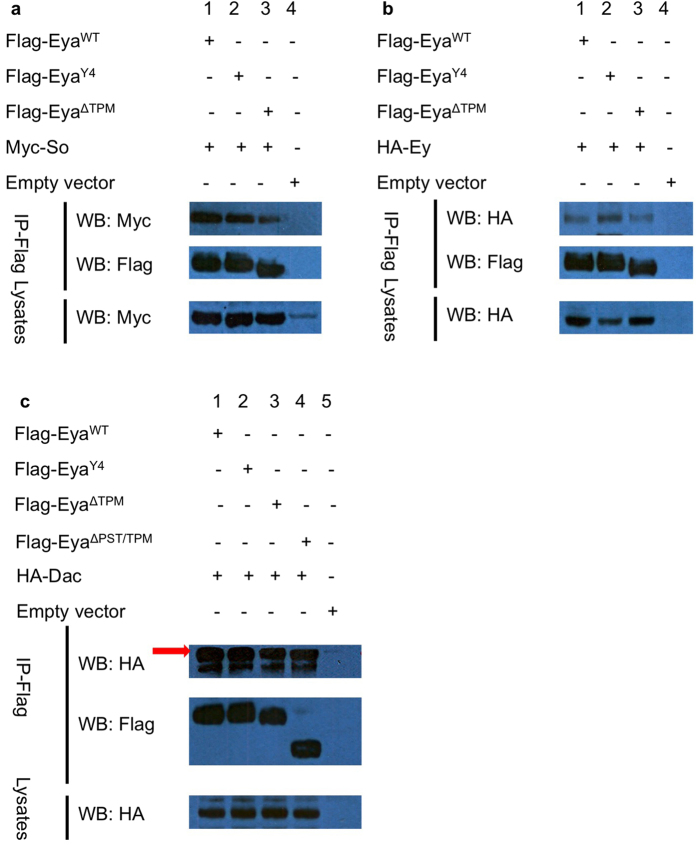
Threonine phosphatase activity is not required for Eya interaction with Ey, So, or Dac. (**a,b**) Co-immunoprecipitation (co-IP) studies between wild-type and threonine phosphatase-dead Eya (Eya^Y4^ and Eya^ΔTPM^) and Ey or So are shown. Flag-tagged Eya was co-expressed with HA-Ey or Myc-So in S2 cells and co-IP with anti-Flag beads followed by western blotting (WB) was performed. Ey, So, and Eya (wild-type and mutants) were detected by anti-HA, anti-Myc, and anti-Flag antibodies, respectively. Lanes 1, 2, and 3 show that Eya^WT^, Eya^Y4^ and Eya^ΔTPM^ can pull down So and Ey, respectively. Empty vector is the negative control (Lane 4). (**c**) co-IP analysis of Eya and Dac. Lane 1, Flag-Eya^WT^/HA-Dac; lane 2, Flag-Eya^Y4^/HA-Dac; lane 3, Flag-Eya^ΔTPM^/HA-Dac; lane 4 Flag-Eya^ΔPST/TPM^/HA-Dac; lane 5, empty vector. Anti-FLAG is used for IP. The red arrow indicates Dac protein. All proteins are expressed at similar levels in crude cell lysates (the bottom panel of each set and data not shown). Western blots presented in a-c were cropped to improve clarity and full-length blots are presented in Supplementary Figs S7–9. All gels were run under the same experimental conditions.

Phosphorylation is well known in other systems to regulate protein complex formation and protein stability via ubiquitin-mediated degradation^33^,^34^. Accordingly, we hypothesized that one or more of the RD proteins are direct substrates for Eya threonine phosphatase and that loss of this activity either disrupts the formation of RD protein complexes and/or destabilizes the RD proteins themselves. Furthermore, this effect may be specific to complexes involving Eyeless (Ey), thereby limiting effects anterior to the MF where Ey is expressed. We tested this hypothesis by doing co-immunoprecipitation (co-IP) in S2 cultured cells transiently transfected with epitope-tagged Eya, Ey, So, and Dac expression constructs. Similar amounts of RD proteins are expressed in transfected cells with or without Eya threonine phosphatase activity, and the Y4 mutation or the TPM deletion do not affect Eya protein expression levels in S2 cells (data not shown). As shown in Fig. 4, Eya^Y4^ and Eya^ΔTPM^ co-IP with Ey, So, and Dac without obviously altered efficiency as wild-type Eya. Notably, this is the first report that Eya can bind to Ey. Previous studies also found that both Eya and Ey proteins interact with So^8^,^35^, suggesting Ey, Eya, and So may form a complex to mediate *Drosophila* eye development. Taken together, these observations suggest that the threonine phosphatase activity of Eya is not essential for interactions with other RD proteins.

### The threonine phosphatase motif of Eya has transcriptional activation function

In addition to threonine phosphatase activity, previous cell culture transactivation reporter assays showed that the TPM has transcriptional activation function^10^. To test the hypothesis that this function is biologically relevant *in vivo*, we replaced the TPM only with VP16, a well-known heterologous transcriptional activation domain (Chasman *et al*., 1989). The resulting construct, *eya*^Δ*TPM*+*VP16*^*GR*, was tested for rescue activity. Remarkably, while *eya*^Δ*TPM*^ can restore about 60% of eye size, VP16 is able to largely complement loss of the TPM and restore eye development to approximately 90% of wild-type, both in *eya*^2^ (Fig. 1a,e,f) and *eya*^*cliIID*^ mutant backgrounds (Fig. S3a). The external eye morphology of *eya*^Δ*TPM*+*VP16*^*GR* rescued eyes shows only minor disorganization compared to *eya*^Δ*TPM*^*GR*.

As shown in Fig. 2f, loss of the TPM causes abnormal ommatidial morphology in adult compound eyes. Flies rescued by one copy of *eya*^Δ*TPM*^*GR* have a reduced number and unusual arrangement of rhabdomeres compared with the normal trapezoidal array of photoreceptors in wild-type animals. Tangential sections of *eya*^2^*; eya*^Δ*TPM*+*VP16*^*GR/*+ adult eyes reveal ommatidia with the correct number and largely normal arrangement of rhabdomeres (Fig. S3b). Moreover, in contrast to wild-type (Fig. S2a) and *eya*^2^*; eya*^Δ*TPM*+*VP16*^*GR/*+ (Fig. S2e) flies, axon terminations in the lamina plexus have irregular gaps and breaks (yellow arrows) in *eya*^Δ*TPM*^*GR* rescued flies, reminiscent of the photoreceptor axon defects in *eya* loss-of-function mutants^36^. These observations suggest that a major role of the TPM during *Drosophila* eye development is to provide transactivation function, that this activity is required for normal ommatidial development and photoreceptor axon projections, and that this function can be largely substituted by the VP16 domain.

### The entire PST/TPM domain of Eya is critical for transcriptional activation during eye development

**Figure 5:**
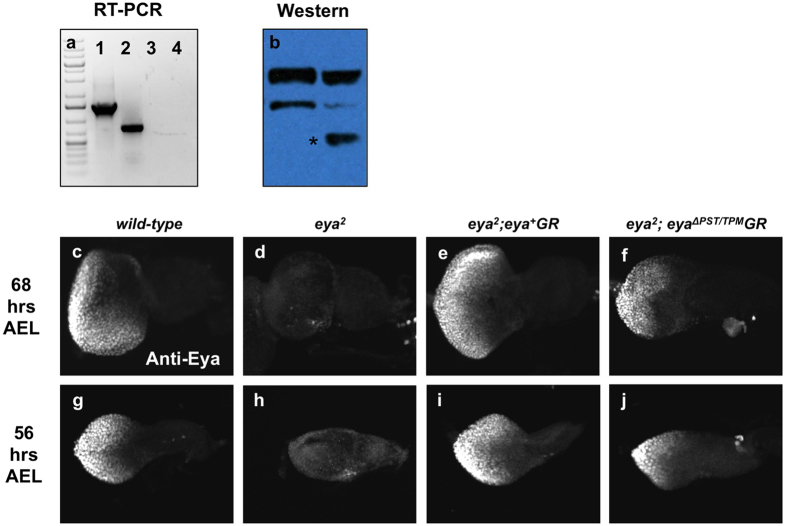
e*ya*^Δ*PST/TPM*^*GR* fails to rescue *eya* mutant defects even though mutant transcript and protein are detected. (**a**) Lanes 1–3 show RT-PCR on RNA prepared from eyes discs 68 hrs after egg laying (AEL). Lane 1: wild-type; Lane 2: *eya*^2^*; eya*^Δ*PST/TPM*^*GR*; Lane 3: *eya*^2^; Lane 4: water. A truncated Δ*PST/TPM* transcript is readily detected (Lane 2). (**b**) An anti-Eya Western blot on extracts prepared from 68 hrs after egg laying (AEL) eye discs (n = 40/lane) from either *eya*^*cliIID*^*/CyO; eya*^+^*GR/*+ or *eya*^*cliIID*^*/CyO; eya*^Δ*PST/TPM*^*GR/*+ animals shows a readily detectable, truncated Δ*PST/TPM* protein (*). Heterozygous *eya*^*cliIID*^ animals were used to obtain enough tissue for the experiment. Western blot presented in b is cropped to improve clarity and full-length blot is presented in Supplementary Fig. S10. (**c–f**) Eye discs prepared from larvae 68 hrs AEL are stained for Eya expression. (**g–j**) Eya staining of 56 hrs AEL eye discs.

The PST/TPM domain of Eya is critical for transactivation in cell culture reporter assays^10^. In order to characterize the *Drosophila* Eya transcriptional activity in its native context *in vivo*, we generated four genomic rescue constructs: *eya*^Δ*PST/TPM*^*GR* (deletion of the PST/TPM domain), *eya*^Δ*PST*^*GR* (deletion of the PST domain alone), *eya*^Δ*PST/TPM* +*VP16*^*GR* (substitution of both the PST and TPM domains with the VP16 activation domain) and eya^Δ*PST*+*VP16*^GR (substitution of the PST domain alone with the VP16 activation domain) (Fig. 1a). We found that *eya*^Δ*PST/TPM*^*GR* completely fails to rescue *eya*^2^ or *eya*^*cliIID*^ mutant phenotypes, even when the transgene is present in two copies (Fig. 1h and data not shown). We can readily detect the predicted, truncated *eya*^Δ*PST/TPM*^ transcript and protein (Fig. 5a–f) in late second instar eye discs prior to MF initiation, suggesting that although the transgene is expressed, at least initially, the Eya^ΔPST/TPM^ protein is non-functional. While the *eya*^Δ*PST/TPM*^*GR* construct completely fails to rescue *eya*^2^ mutant animals, the *eya*^Δ*PST*^*GR* retains slightly more function and can rescue about 5% of normal eye size (Fig. 1j). Previous S2 cell culture studies have suggested that both the PST and TPM domains contribute transcription activation function^10^ and our GR data are consistent with these results. In addition to *eya*^Δ*TPM*+*VP16*^*GR*, our other VP16 substitution genomic rescue results also confirm these findings. Specifically, the *eya*^Δ*PST/TPM* +*VP16*^*GR* is sufficient to rescue about 5% of eye size in an *eya*^2^ background (Fig. 1i), similar to that of the *eya*^Δ*PST*^*GR* construct alone. *eya*^Δ*PST*+*VP16*^*GR* is able to restore eye development to ~30% of wild-type (Fig. 1k). Two copies of *eya*^Δ*PST/TPM* +*VP16*^*GR* or *eya*^Δ*PST*+*VP16*^*GR* consistently rescue *eya*^2^ eye size better than one copy (Fig. S4). Moreover, *eya*^Δ*PST/TPM* +*VP16*^*GR*, *eya*^Δ*PST*^*GR*, and *eya*^Δ*PST*+*VP16*^*GR* fail to rescue *eya*^*cliIID*^ mutants. These functional dissection studies reveal that the transactivation domain PST/TPM is essential for eye formation and viability in *Drosophila*. In addition, the PST domain is likely playing a more significant role than the TPM during *Drosophila* development since *eya*^Δ*TPM*^*GR* rescues 60% of the eye size compared to 5% of the eye size rescued by *eya*^Δ*PST*^*GR* and *eya*^Δ*TPM*^*GR* is able to restores viability to *eya*^*cliIID*^ null mutants.

### The PST/TPM domain regulates retinal determination gene expression

**Figure 6:**
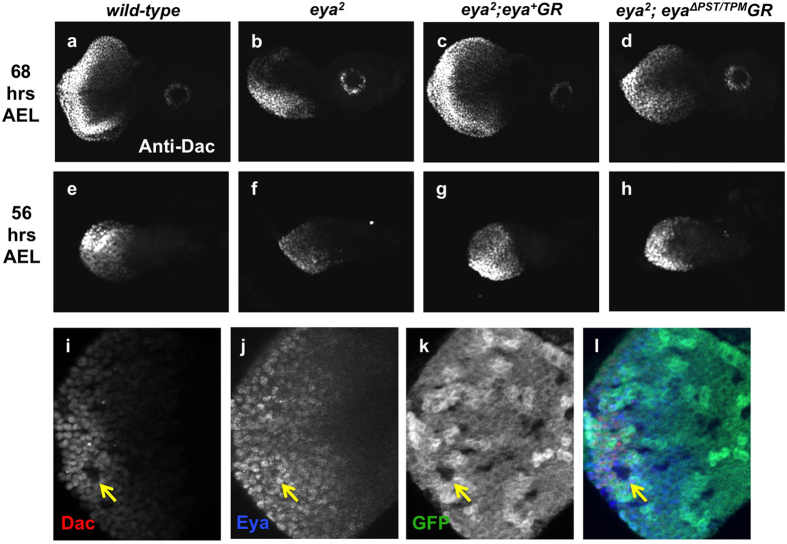
PST/TPM is required for normal Dac expression. (**a–d**) Dac expression in *Canton-S* (**a**), *eya*^2^ (**b**), *eya*^2^*; eya*^+^*GR* (**c**) and *eya*^2^*; eya*^Δ*PST/TPM*^*GR* (**d**) eye imaginal discs from 68 hrs AEL. (**e–h**) Immunostaining of Dac on 56 hrs AEL eye discs. (**i–k**) Dac, Eya and GFP expression in *eya*^Δ*PST/TPM*^ rescued *eya*^*cliIID*^ null clones. Yellow arrow indicates one of the larger, more posterior clones in which Dac expression is reduced. (**l**) Merge of channels.

Eya can act as a transcriptional coactivator and physically interact with other RD proteins to regulate multiple developmental processes^7^,^8^,^9^,^10^,^37^. Therefore, we were interested in understanding the role of PST/TPM in RD gene regulation since it is critical for Eya function. Since *eya*^Δ*PST/TPM*^*GR* fails to rescue the eye phenotype of *eya*^2^ animals and little Eya expression is detected at late third instar (data not shown), we used second instar larvae to assess the function of the PST/TPM when Eya^ΔPST/TPM^ protein is still expressed (Fig. 5f). *eya*^2^ flies rescued with two copies of *eya*^Δ*PST/TPM*^*GR* show slightly lower Eya expression compared to wild-type or *eya GR*-rescued animals at 68 hrs after egg laying (AEL) (Fig. 5c–f). We also found that Eya expression in *eya*^2^*; eya*^Δ*PST/TPM*^*GR* eye discs is lower than that of wild-type discs at 56 hrs AEL (Fig. 5g–j). Similar reductions are observed for the expression of the retinal determination protein Dac, a known downstream target of Eya (Fig. 6a–h). In addition, in *eya*^Δ*PST/TPM*^ clones (*eya*^*cliIID*^ null clones rescued by a single copy of *eya*^Δ*PST/TPM*^*GR*) at 72 hrs AEL, Dac expression is reduced while the expression of Eya^ΔPST/TPM^ is normal (Fig. 6i–l, yellow arrows). Taken together, these data imply that the PST/TPM domain of Eya is required for normal Dac expression.

**Figure 7:**
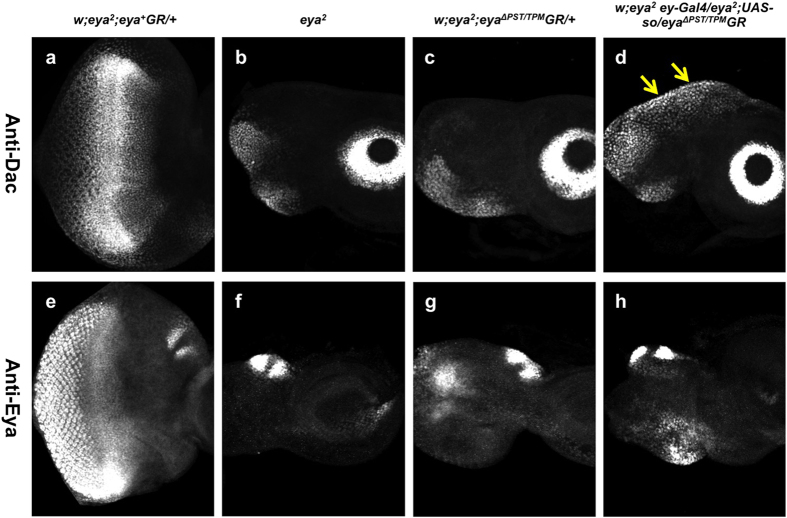
Overexpression of So in *eya*^2^; *eya*^Δ*PST/TPM*^*GR* animals causes increased Dac expression. (**a–d**) Dac staining in third instar larvae after inducing *so* expression with *ey-Gal4/UAS-so*. Yellow arrows (**d**) indicate region of the disc in which Dac expression is increased. (**e–h**) Eya expression after *ey-Gal4/UAS-so* induction. *eya*^2^*; eya*^+^*GR* (**a,e**) and *eya*^2^ (**b,f**) are used as positive and negative controls.

Moreover, *ey-Gal4* induced So expression in *eya*^2^ animals rescued by one copy of eya^Δ*PST/TPM*^*GR* partially restores Dac expression (Fig. 7a–d), but has no effect on expression of Eya (Fig. 7e–h). These observations suggest that the PST/TPM positively regulates expression of Dac through the Eya binding partner So.

**Figure 8:**
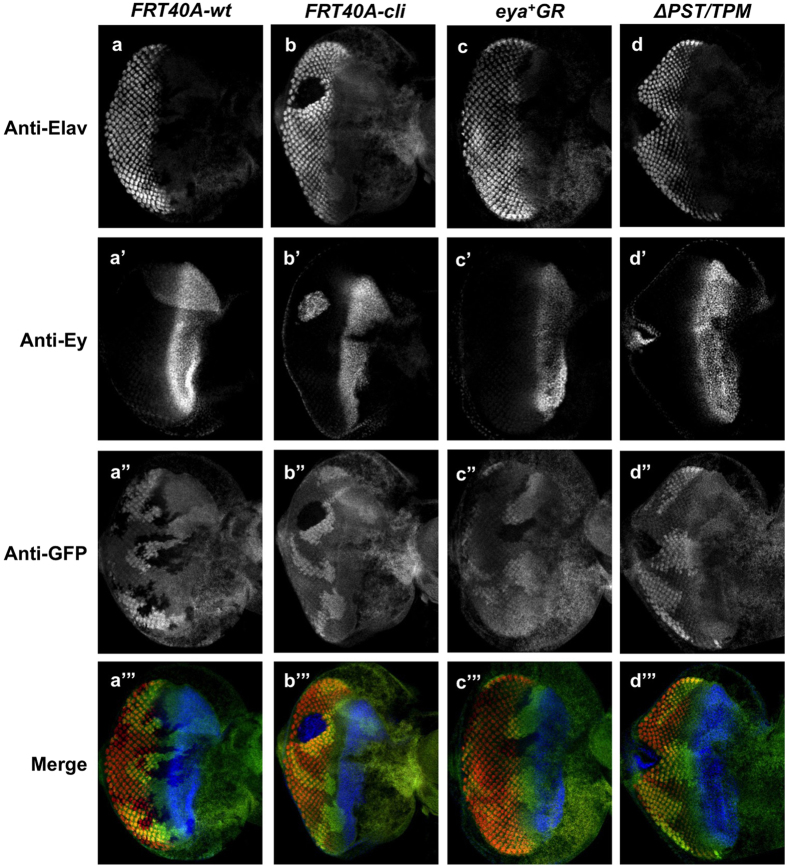
The PST/TPM of Eya is necessary for *ey* repression posterior to the morphogenetic furrow. (**a–a’”**) Wild-type clones. (**b–b’”**) *eya*^*cliIID*^ null clones. (**c–c’”**) *eya*^+^*GR* rescued *eya*^*cliIID*^ null clones. (**d–d’”**) *eya*^Δ*PST/TPM*^*GR* rescued *eya*^*cliIID*^ null clones. Grayscale images of Elav, Ey, and GFP expression are shown in grayscale (**a–d”**) and as red, blue, and green, respectively, in a’”–d’”; Elav marks differentiating photoreceptors and complete loss of GFP expression marks homozygous mutant clones.

To test if the PST/TPM deletion affects Ey regulation and photoreceptor differentiation, we assayed Ey and Elav expression in *eya*^Δ*PST/TPM*^ rescued *eya* null mutant clones. We found that *eya*^Δ*PST/TPM*^ clones show a complete loss of Elav expression, a marker of photoreceptor differentiation^38^, posterior to the MF (Fig. 8a–d). In *eya*^Δ*PST/TPM*^ clones posterior to the furrow, we found strong Ey expression (Fig. 8a’–d’), suggesting the PST/TPM domain of Eya is required for Ey suppression. Additionally, *eya*^Δ*PST/TPM*^ clones result in the loss of photoreceptor development and black overgrowths in adults (Fig. S5d).

### Deletion of the PST/TPM does not abolish interactions between Eya and Ey, So, or Dac

**Figure 9:**
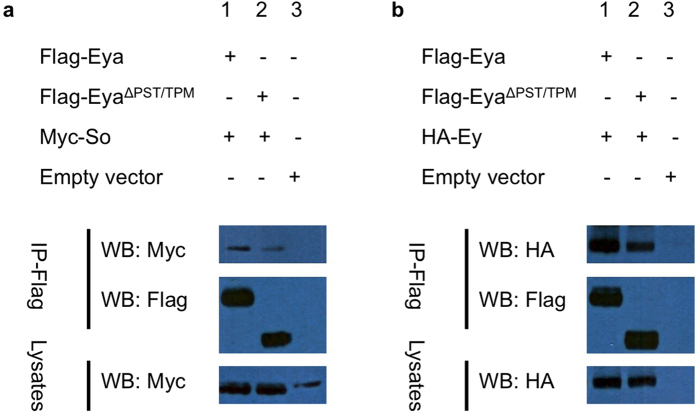
The PST/TPM domain is not required for interaction between Eya and Ey, So, or Dac. (**a,b**) S2 cells were transfected as described in Materials and Methods, lysates were immunoprecipitated (IP) with anti-Flag beads, and then immunoblotted (WB) with anti-Myc, anti-HA, and anti-Flag antibodies to detect Myc-So, HA-Ey, and Flag-Eya, respectively. Co-IP for Eya^ΔPST/TPM^ and Dac is shown in Fig. 4c. Western blots presented in a-b were cropped to improve clarity and full-length blots are presented in Supplementary Fig. S11–12. All gels were run under the same experimental conditions.

So and Dac are known binding partners of Eya^7^,^8^,^11^. Since *eya*^Δ*PST/TPM*^*GR* rescued flies have no eyes, similar to the loss-of-function phenotypes of the core RD genes (*ey*, *so*, and *dac*), we hypothesized that the PST/TPM domain may mediate specific, essential interactions between Eya and Ey, So, or Dac. To test this hypothesis, we carried out co-immunoprecipitation (co-IP) experiments. As shown in Figs 4 and 9, both wild-type and Eya^ΔPST/TPM^ can co-IP with Ey, So, and Dac, suggesting that deletion of the PST/TPM does not abolish the interactions between Eya and these three RD proteins. These observations are consistent with previous findings that Eya-So and Eya-Dac interaction is mediated via the ED of Eya^7^,^8^,^11^. The Eya domain that mediates Eya-Ey physical interaction remains to be determined.

## Discussion

In this paper we report that loss of threonine phosphatase activity has little effect on *Drosophila* eye development, since eye development in *eya*^*Y4*^*GR* rescued flies proceeds relatively normally. On the other hand, the essential function of the PST and the threonine phosphatase motif (TPM) is transcriptional activation that can be largely complemented by the heterologous activation domain VP16. Together with our findings that the PST and TPM are required for normal *Drosophila* eye development, we conclude that a major function of Eya during *Drosophila* eye development is as a transcriptional coactivator. Although the tyrosine phosphatase activity of the Eya Domain (ED) is dispensable for Eya function^25^, the specific role the ED plays *in vivo* has not been reported.

The retinal determination (RD) network is a small group of highly conserved transcriptional regulators that are both necessary for eye development and sufficient to trigger ectopic eye formation when overexpressed in other imaginal discs^1^,^2^,^3^,^4^,^5^,^6^,^7^,^8^,^14^,^21^,^39^. As a vital member of the RD network, a unique feature of the Eya proteins is that they have several distinct biochemical activities. In *Drosophila*, previous cell culture reporter assays and cDNA-based *Gal4-UAS* genetic rescue studies suggested that the PST-rich region is a transactivation domain and plays a role in ectopic eye induction, while the TPM and ED possess threonine and tyrosine phosphatase activity, respectively^10^,^18^,^19^,^24^. Intriguingly, our results using genomic rescue constructs are consistent with previous studies of the PST/TPM transactivation domain, but are contrary to previous reports that the tyrosine phosphatase domain, but not the threonine phosphatase domain, governs *Drosophila* eye development.

In our work, we have found that both the TPM and PST contribute transcriptional activation for normal eye development. Substituting the heterologous activation domain VP16 for the TPM and PST domain substantially restores Eya function. Two reasons could account for the failure of complete rescue by VP16. First, the TPM or PST have other, distinct functions. Although we have excluded the possibility that the TPM and PST are required for Eya binding with Ey, So, or Dac in this report, we cannot rule out other possibilities. For example, previous findings identified the PST/TPM domain of Eya as the primary target of Nmo and Abl-mediated phosphorylation in kinase assays^36^,^40^. Second, there may be insufficient activation function provided by VP16 - perhaps due to an inability to make specific contacts with other proteins, or that the fusion proteins do not have the proper conformation to interact properly via other domains.

The transcriptional role of Eya has been studied in *Drosophila* through genetic and/or biochemical interaction with the transcription factors So and Dac^7^,^8^. In this paper, we further indicate that the PST/TPM domain positively regulates Dac expression and this regulation may be mediated via So. Moreover, the PST/TPM is required to suppress Ey expression posterior to the furrow. These observations are consistent with previous reports that *dac* expression requires both *so* and *eya*^7^,^14^,^39^,^41^ and both Eya and So are necessary to mediate Ey repression posterior to the MF^42^. Our studies localize these functions of Eya to the PST/TPM domain.

Although genetic interactions between Eya and Ey have been widely reported, physical interactions between these two RD proteins have not. In this paper, we report that Eya physically interacts with Ey for the first time. Previous studies also found physical interactions between Eya-So^8^ and Ey-So^35^, suggesting that Ey-Eya-So may form a ternary complex. In addition, previous findings show that ectopic eye induction by Ey requires the presence of Eya and So^43^, and the expression patterns of all three genes overlap extensively and are nearly identical anterior to the MF^43^. Moreover, misexpression of Eya and So induces the formation of ectopic eyes; however, this effect is lost in an *ey* mutant background^8^,^21^. Finally, *ey* is a direct target of Eya and So^11^,^44^ and vice versa - *eya* and *so* are direct targets of Ey^45^,^46^. Since Groucho is a repressor of the Eya-So complex^10^, Ey may act as an activator of Eya-So to increase transcriptional output of Dac. Consistent with this hypothesis, loss of *ey*, *eya*, or *so* function causes loss of Dac expression, suggesting that Ey, So, and Eya are primary regulators of Dac^7^,^8^,^47^. Similar relationships have been observed with *Pax6*, *Eya1/2* and *Six3*, mouse orthologs of *ey, eya*, and *so*, respectively. Specifically, mouse *Pax6* mutants have reduced levels of *Eya1* and *eya*^2^ in the optic vesicle and overlying ectoderm^48^,^49^ and Pax6 induces expression of *Six3* when ectopically expressed in mice^50^. In addition, we used STRING^51^, a database of known and predicted protein interactions, to predict protein-protein interactions for Ey, Eya and So. As expected, we found equally high associations for all three pairs of complexes (Fig. S6), providing further evidence of strong interactions among these RD proteins, which may act together in a ternary complex.

In addition, our genomic rescue assays show that the threonine phosphatase activity is largely but not entirely dispensable for *Drosophila* eye development. Our threonine-phosphatase inactive GRs can robustly rescue eye formation in *eya* null mutants, but the rescued eyes show disorganized external and internal morphology as compared to wild-type rescue controls. This result is in contrast to another report based on the *GAL4-UAS* system that finds the threonine phosphatase activity of Eya to be dispensable during eye development^24^. The reason for this difference is that our GR system offers higher resolution thereby allowing detection of more subtle defects in morphology, while the *GAL4-UAS* system is a less accurate approach. In particular, Liu *et al*. did in fact observe a disorganized eye phenotype in *eya*^2^ flies rescued by *UAS-eya*^*Y4*^. However, this phenotype appeared similar to the imperfect rescue achieved with the wild-type *UAS-eya* transgene. For this reason, they could not uncover the requirement for the threonine phosphatase activity during differentiation. This report highlights the need for careful interpretation of results based on the *GAL4-UAS* system and the superior sensitivity of the GR method. Although the threonine phosphatase activity of Eya plays only a minor role during eye development, it has been reported to be involved in the innate immune response in both *Drosophila* and mouse^17^,^24^.

In summary, we have shown that both the transcriptional activation and threonine phosphatase activity of Eya are required for normal *Drosophila* eye development. However, a primary function of Eya during this process is transcriptional coactivation, while the phosphatase activity plays only a minor role. Our study provides an accurate approach to assess the functional significance of individual protein domains *in vivo*, highlighting the importance of the transactivation function of Eya during *Drosophila* development. As Eya is conserved and plays important roles in retinal development throughout the metazoa, the underlying mechanisms of Eya function are likely to be conserved in vertebrates as well.

## Methods

### Fly strains and maintenance

All flies were maintained with standard corn meal and yeast extract medium at 25 °C. *Canton-S* was used as a wild-type control. Heat shocks were performed at 37 °C as described previously^52^. To test the function of the mutant *eyaGR* during eye development, we crossed transgenes into the following mutant backgrounds: *eya*^2^, which completely lack eyes due to a deletion of an enhancer required for *eya* expression during eye development^4^, and *eya*^*cliIID*^, which is a null allele caused by a premature stop codon that causes recessive embryonic lethality^53^. Wild-type clones and *eya*^Δ*PST/TPM*^ clones were generated by crossing *w/Y; FRT40A* and *w/Y; eya*^*clillD*^ *FRT40A/CyO; eya*^Δ*PST/TPM*^*GR* with *ywhs-flp; w*+*ubiGFP, FRT40A* animals, respectively.

### Recombineering-induced mutagenesis of *eya* ^+^ *GR* and *Drosophila* transgenesis

A two-step recombineering method was used to create the Y4, ΔTPM, ΔTPM+VP16, ΔPST/TPM, ΔPST/TPM+VP16, ΔPST and ΔPST+VP16 mutations in the *eya*^+^*GR* construct as described previously^54^. Recombineering products were verified by DNA sequencing and restriction enzyme fingerprint digestion prior to transgenesis. Constructs were inserted into the *attP2* docking site on the third chromosome using PhiC31-mediated transgenesis and site-specific integration was confirmed by genomic PCR with attP/attB primers^28^. Transgenic flies were confirmed by genomic DNA PCR sequencing. Primer sequences are available on request.

### Construction of cell culture expression plasmids

We used the Q5 Site-Directed Mutagenesis Kit (NEB) to introduce a series of mutations in cell culture expression plasmids which were confirmed by DNA sequencing. These mutations include: pMT-Flag-Eya^Y4^, pMT-Flag-Eya^ΔTPM^, pMT-Flag-Eya^ΔPST/TPM^ and pMT-HA-Dac. pAHW-Ey was generated from destination vector pAHW and pUAST-Ey (a gift from Dr. Rui Chen, Houston, TX) according to the Gateway protocol provided by the *Drosophila* Genomics Resource Center. pMT-Flag-Eya, pMT-Myc-So, pMT-dac, and pAHW were kindly provided by Dr. Ilaria Rebay (Chicago, IL). Primers used in this report are listed in Table S1.

### S2 cell culture and transfection

*Drosophila* S2 cells were cultured in Schneider’s medium containing 10% heat-inactivated fetal bovine serum and antibiotics at 25 °C. Cells were transiently transfected in 6-well plates using the FuGENE HD Transfection Reagent (Promega) according to the manufacturer’s protocol. 24 hrs after transfection, cells were induced by addition of 0.1 M CuSO4.

### Co-IP and western blots

Transfected cells were lysed by rocking at 4 °C for 30 min in Pierce IP lysis buffer (Thermo Fisher Scientific) with a Roche Complete, Mini, EDTA-free protease inhibitor cocktail tablet. The lysates were subjected to immunoprecipitation with anti-Flag-conjugated agarose beads (Sigma) for 2 h at 4 °C. After washing three times with lysis buffer, immunoprecipitates were boiled in 4× NuPAGE LDS sample buffer (Novex), and western blotting was carried out according to the NuPAGE electrophoresis (Novex) protocol with rabbit anti-Flag (1:1000, Sigma), rabbit anti-MYC (1:100, Santa Cruz Biotechnology), and rabbit anti-HA (1:200, Santa Cruz Biotechnology) antibodies.

For tissue preparation, 68 hrs AEL eye discs (n = 40) were collected in cold RIPA lysis buffer (Thermo Fisher Scientific). After centrifuge at 20000 g for 10 min at 4 °C, the supernatant was transferred to a new tube and ready for western blot analysis.

### Histology and immunohistochemistry

Staining of eye discs and imaging of the adult eye were conducted as described previously^42^. Immunohistochemistry on 48 hr pupal eye discs and tangential sections of adult eyes were generated as previously described^55^. For antibodies used, please reference Table S2.

### RT-PCR

RNA was extracted from 56 hrs AEL eye discs using PureLink RNA Mini Kit (Ambion). Reverse transcription was performed according to the instructions of SuperScript One-Step RT-PCR kit (Invitrogen).

## Additional Information

**How to cite this article**: Jin, M. and Mardon, G. Distinct Biochemical Activities of Eyes absent During *Drosophila* Eye Development. *Sci. Rep*. **6**, 23228; doi: 10.1038/srep23228 (2016).

## Supplementary Material

Supplementary Information
