# Supplementary material for: Distinct Biochemical Activities of Eyes absent During Drosophila Eye Development
Source: Sci Rep. 2016 Mar 16;6:23228. doi: 10.1038/srep23228 (PMC4793267; doi:10.1038/srep23228)
Supplement: Supplementary Information [file srep23228-s1.pdf]

Supplementary information

Distinct Biochemical Activities of Eyes absent During *Drosophila* Eye Development

Meng Jin[1] and Graeme Mardon[1-6]*

[1]Department of Pathology and Immunology, Baylor College of Medicine, Houston, TX 77030, USA
[2]Program in Developmental Biology, Baylor College of Medicine, Houston, TX 77030, USA
[3]Department of Molecular and Human Genetics, Baylor College of Medicine, Houston, TX 77030, USA
[4]Department of Neuroscience, Baylor College of Medicine, Houston, TX 77030, USA
[5]Department of Ophthalmology, Baylor College of Medicine, Houston, TX 77030, USA
[6]Program in Cell and Molecular Biology, Baylor College of Medicine, One Baylor Plaza, Houston, TX 77030, USA

*Corresponding author
Email: gmardon@bcm.edu

Supplementary Fig. S1

eya^{cliIID}; $eya^{Y4}GR/+$ ***eya^{cliIID}; $eya^{\Delta TPM}GR/+$***

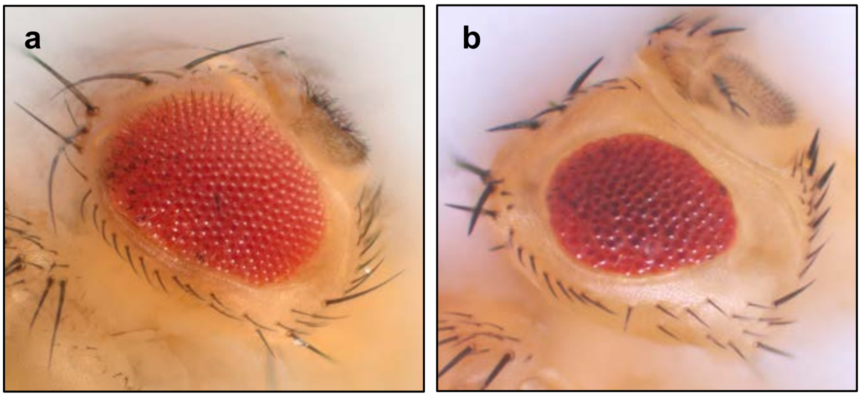

Supplementary Fig. S1. The threonine phosphatase activity is not required for viability. Adult eyes of *eya^{cliIID}* mutant flies rescued by one copy of *$eya^{Y4}GR$* (**a**) and *$eya^{\Delta TPM}GR$* (**b**).

Supplementary Fig. S2

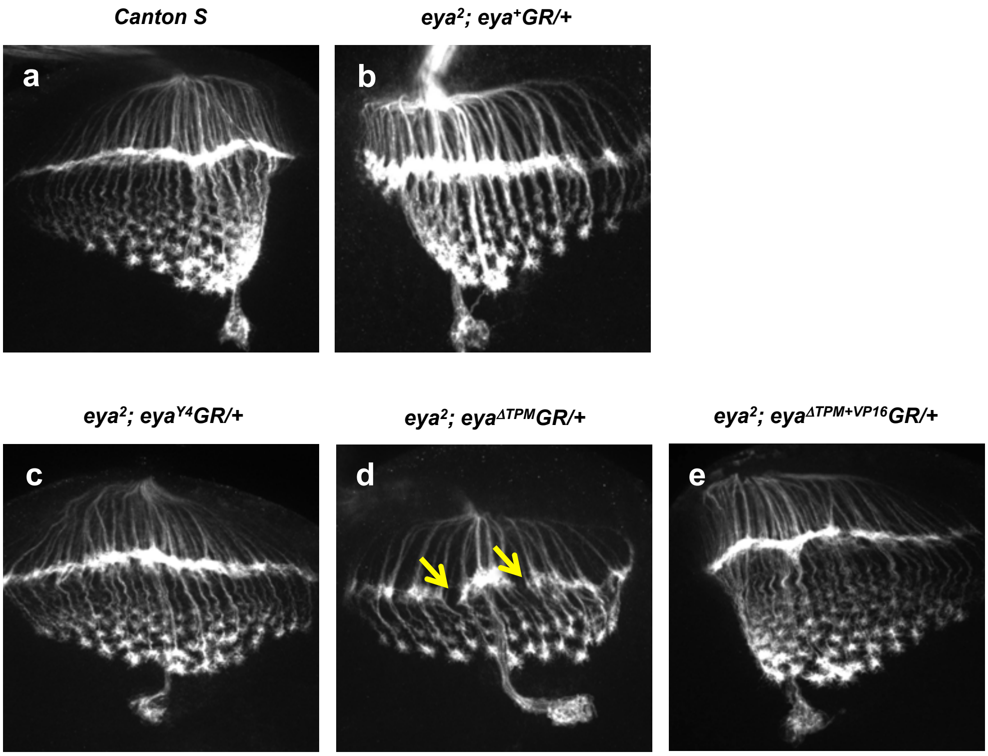

Supplementary Fig. S2. VP16 complements loss of the TPM and restores photoreceptor axon projections. Projections from photoreceptors R1–8 of *Canton S* (**a**), *eya^2; $eya^+GR/+$* (**b**), *eya^2; $eya^{Y4}GR$* (**c**), *eya^2; $eya^{\Delta TPM}GR$* (**d**) and *eya^2; $eya^{\Delta TPM+VP16}GR$* (**e**) are visualized with anti-Chaoptin. Arrows indicate irregular lamina plexus breaks.

Supplementary Fig. S3

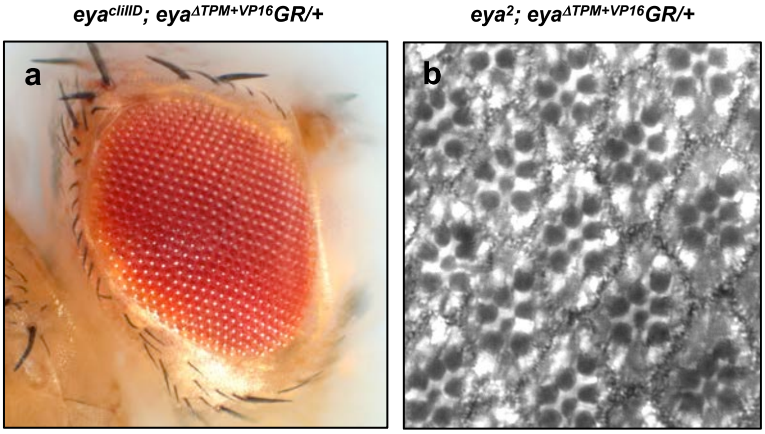

Supplementary Fig. S3. VP16 largely complements loss of the TPM and restores eye development. External (**a**) and internal (**b**) eye morphology of *eya* mutant flies rescued by one copy of $eya^{\Delta TPM+VP16}GR$.

Supplementary Fig. S4

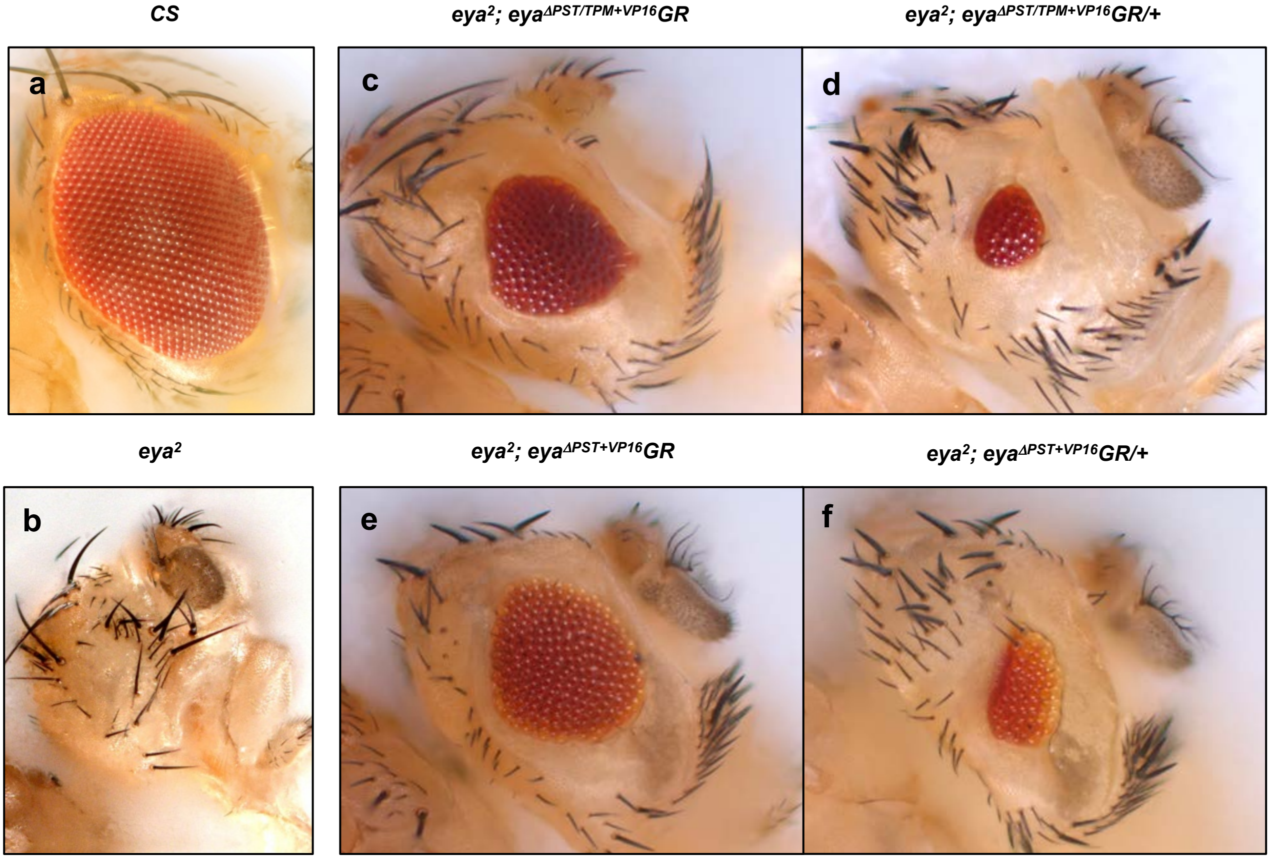

Supplementary Fig. S4. Two copies of *eya*$^{\Delta PST/TPM+VP16}$*GR* or *eya*$^{\Delta PST+VP16}$*GR* rescue *eya*^{2} eye size better than one copy. Adult eyes of *Canton S* (**a**), *eya*^{2} (**b**), *eya*^{2} flies rescued with one or two copies of *eya*$^{\Delta PST/TPM+VP16}$*GR* (**c,d**) and *eya*^{2} flies rescued with one or two copies of *eya*$^{\Delta PST+VP16}$*GR* (**e,f**).

Supplementary Fig. S5

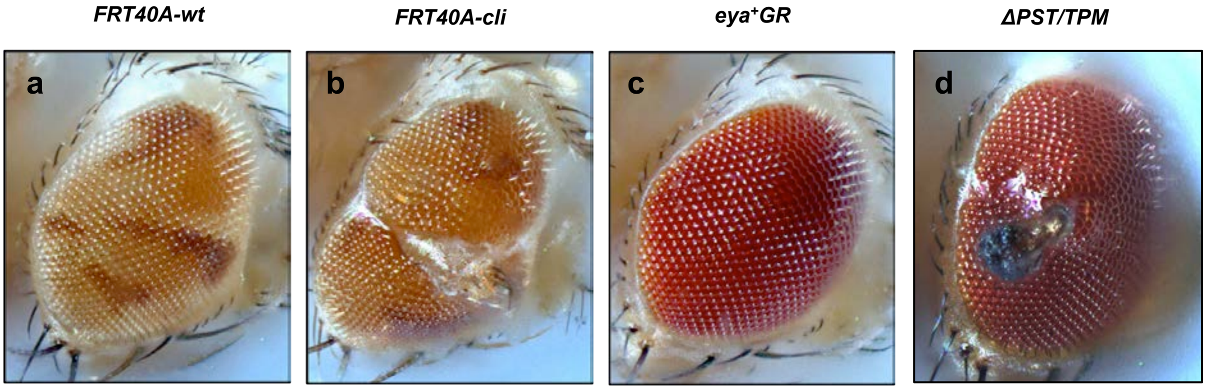

Supplementary Fig. S5. Adult eyes of *eya^{cliIID}* null clones rescued by *$eya^{\Delta PST/TPM}GR$*. Adult eyes of wild-type clones (**a**), *eya^{cliIID}* null clones (**b**), *$eya^{+}GR$* rescued *eya^{cliIID}* null clones (**c**), and *$eya^{\Delta PST/TPM}GR$* rescued *eya^{cliIID}* null clones (**d**).

Supplementary Fig. S6

a

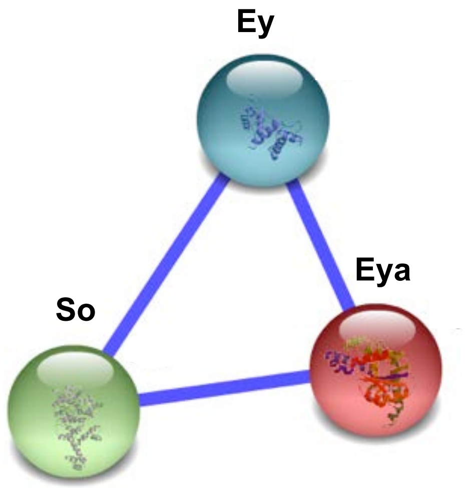

b

Node1	Node2	Experimental	Textmining	Combined_score
**Ey**	**So**	0.885	0.850	0.982
**So**	**Eya**	0.999	0.991	0.999
**Ey**	**Eya**	0.992	0.982	0.999

Supplementary Fig. S6. Protein-protein interactions among Ey, Eya and So. (**a**) The interaction map shown was generated based on the STRING database. The interactions include direct (physical) and indirect (functional) associations derived from experimental assays and literature mining. (**b**) Association scores based on different sources of interaction evidence. Confidence range (low confidence: scores <0.4; medium: 0.4 to 0.7; high: >0.7).

Supplementary Fig. S7

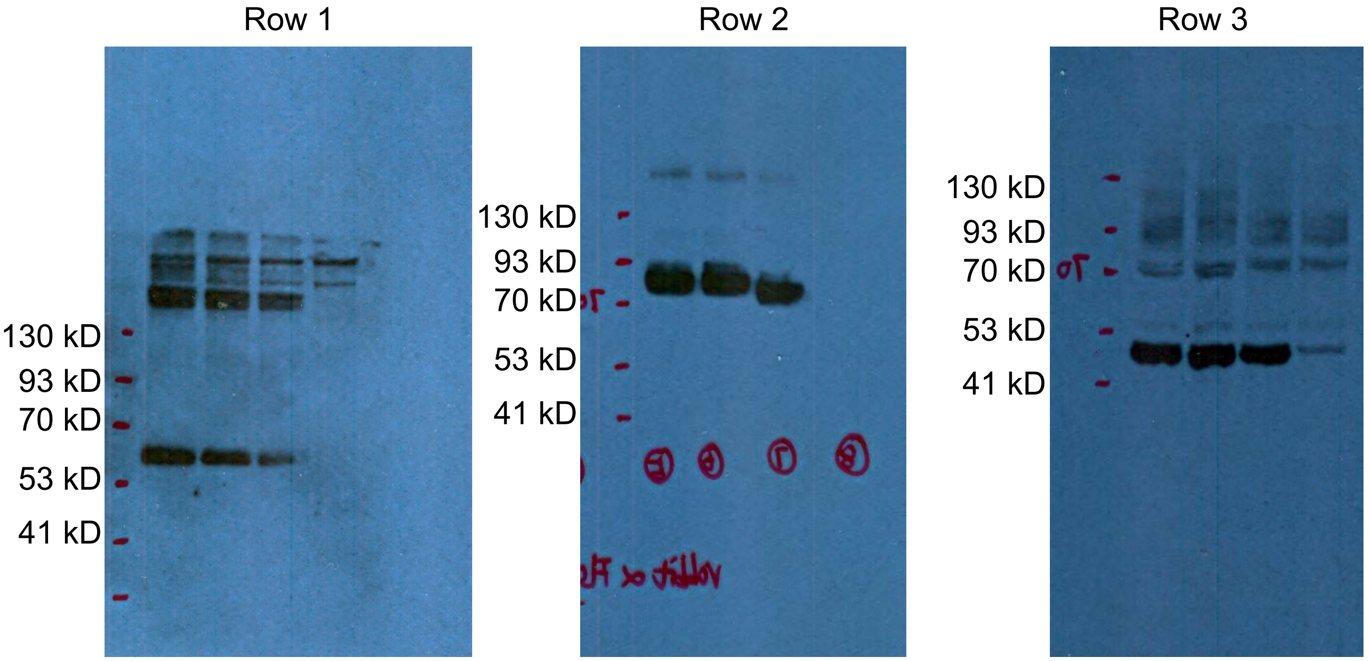

Supplementary Fig. S7. Full-length blots of Fig. 4a

Supplementary Fig. S8

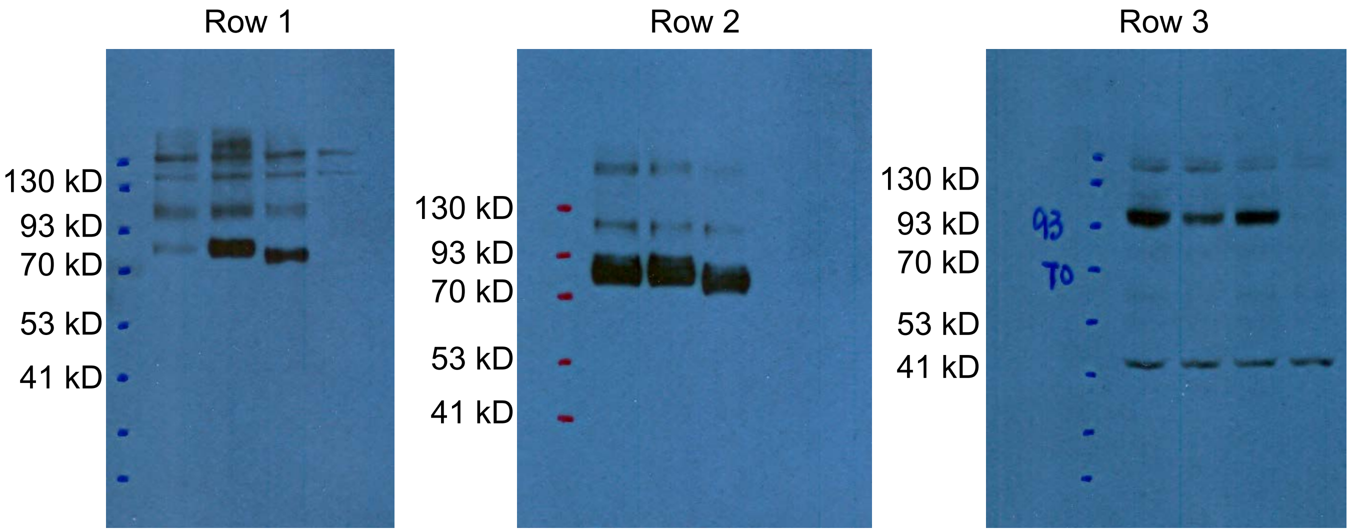

Supplementary Fig. S8. Full-length blots of Fig. 4b

Supplementary Fig. S9

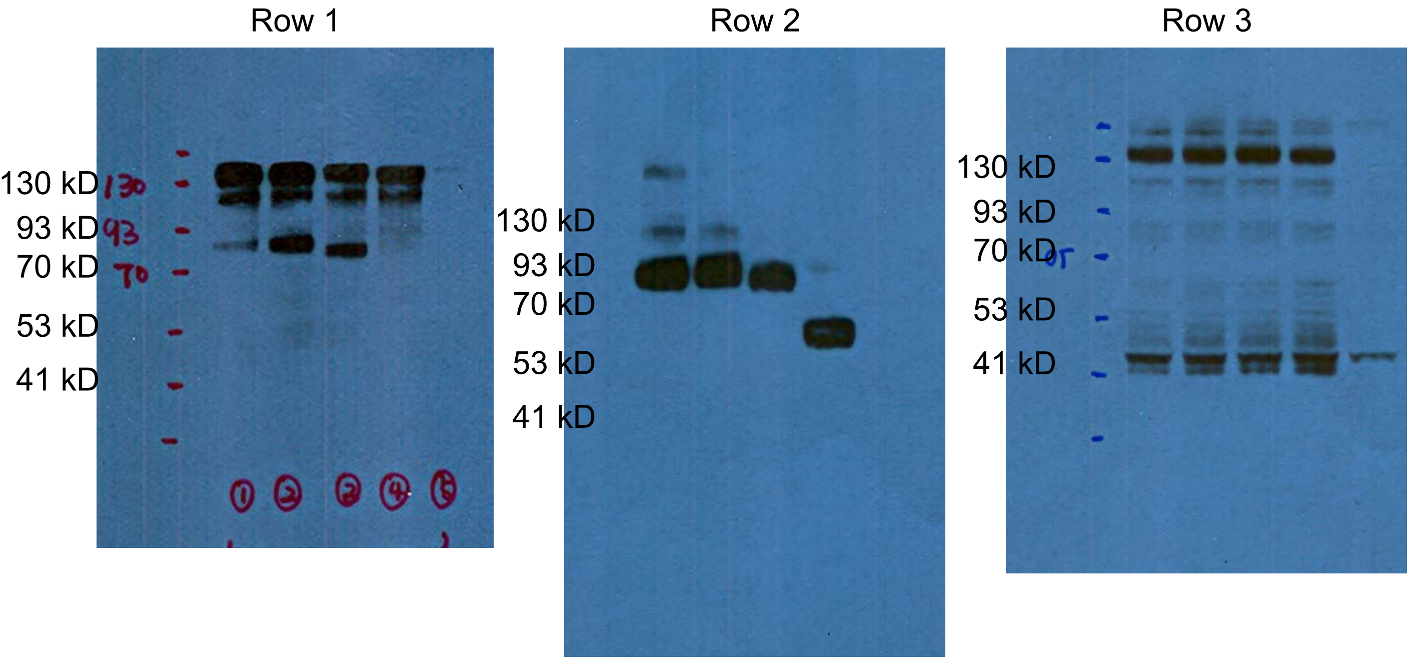

Supplementary Fig. S9. Full-length blots of Fig. 4c

Supplementary Fig. S10

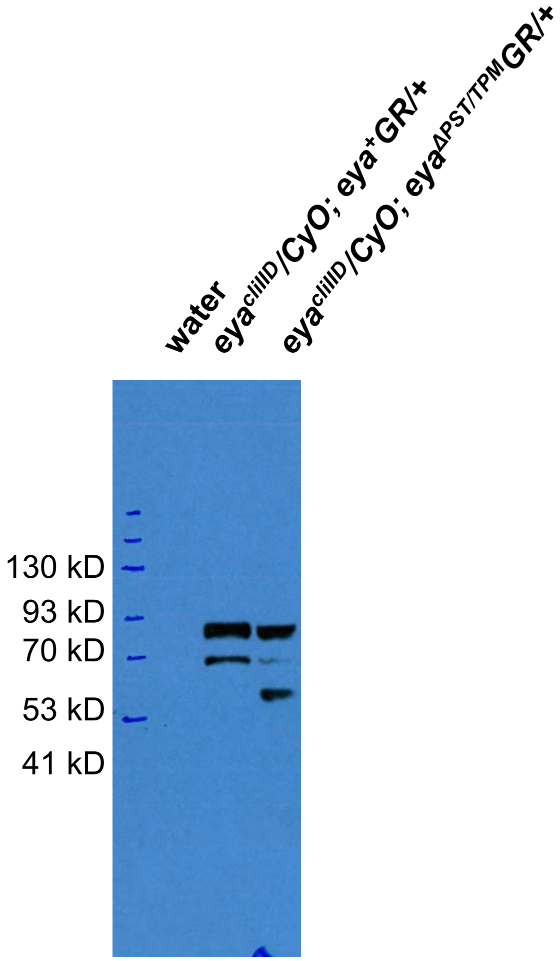

Supplementary Fig. S10. Full-length blot of Fig. 5b

Supplementary Fig. S11

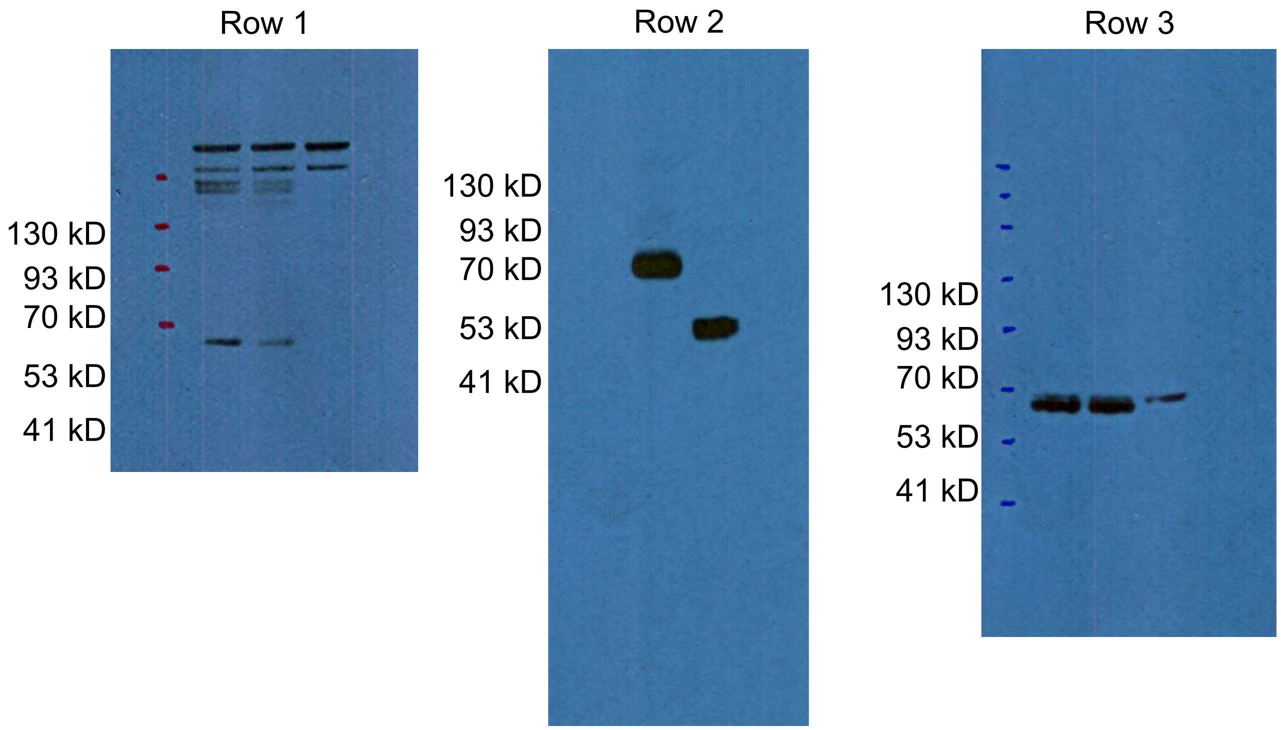

Supplementary Fig. S11. Full-length blots of Fig. 9a

Supplementary Fig. S12

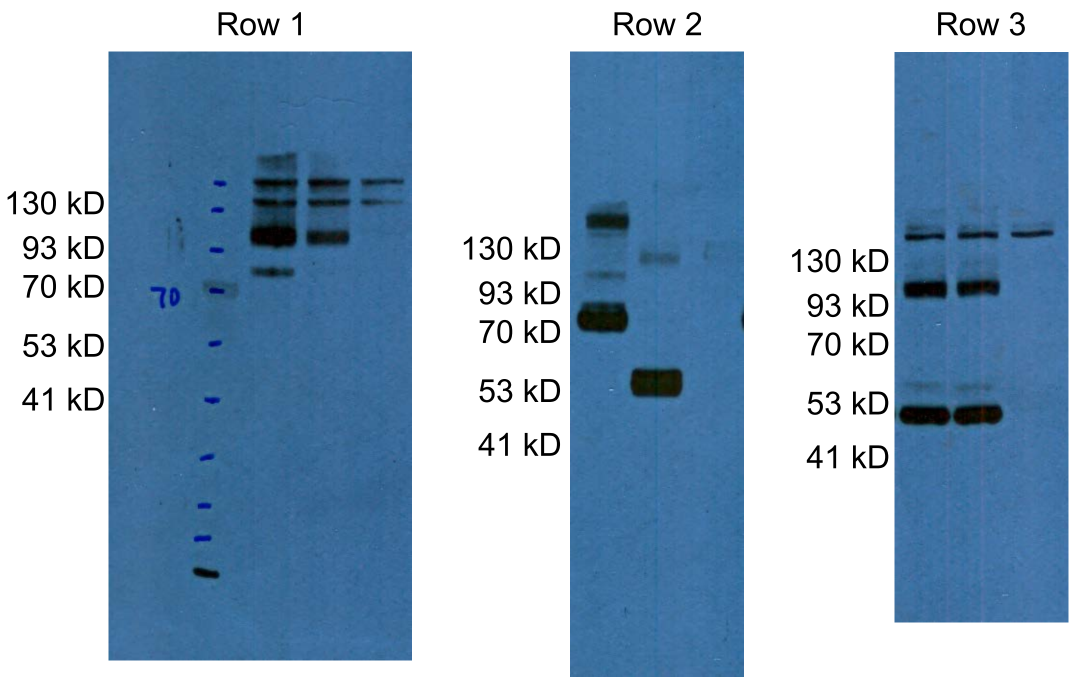

Supplementary Fig. S12. Full-length blots of Fig. 9b

Supplementary Table S1. Primers used in expression plasmids.

**Primer Name**	**Template**	**Sequence**
Flag-Y4-Q5F	pMT-Flag-Eya	AGTCCGGCCAACGCCTCACCGTATGCGGTCAGC
Flag-Y4-Q5R	pMT-Flag-Eya	GGCAGCGTTGCCGGCCTGCTCGTTGTAGTAGCC
Flag-ΔTPM-Q5-F	pMT-Flag-Eya	TATGCGGTCAGCTCGCCC
Flag-ΔTPM-Q5-R	pMT-Flag-Eya	GTTGTAGCCGGCGTACGG
Flag-ΔPST/TPM-Q5-F	pMT-Flag-Eya	ACCGCCGGCTCTGGGGGC
Flag-ΔPST/TPM-Q5-F	pMT-Flag-Eya	GGCCACCGAGCCGTTGTCCAG
ey-ORF-F	pUAST-Ey	CACCATGTTTACATTGCAACCAACTCCA
ey-ORF-R	pUAST-Ey	CTAGACCCACGGTGAGTAGAAAC
HA-dac-Q5-F	pMT-dac	CCGGACTATGCAGGATCCTATCCATATGACGTTCC AGATTACGCTGATTCTGTGACAAGTGAAC
HA-dac-Q5-R	pMT-dac	GACGTCATAGGGATAGCCCGCATAGTCAGGAACA TCGTATGGGTACATACTTCATCATGGCCG

Supplementary Table S2. Antibodies used in immunohistochemistry. Antigen, host, dilution and source are indicated

Antigen	Host	Dilution	Source
Eyes absent	Mouse	1:100	DSHB
Dachshund	Mouse	1:250	DSHB
Cyclin B	Mouse	1:200	DSHB
Dlg	Mouse	1:150	DSHB
Chaoptin	Mouse	1:100	DSHB
Cut	Mouse	1:100	DSHB
ELAV	Rat	1:500	DSHB
Eyeless	Rabbit	1:2500	Uwe Walldorf
GFP	Chicken	1:1000	Abcam
Atonal	Guinea Pig	1:1000	Hugo Bellen
Sine oculis	Guinea Pig	1:2000	Ilaria Rebay
Rabbit IgG, Alexa 488 conjugated	Goat	1:600	Molecular Probes
Rabbit IgG, Cy5 conjugated	Goat	1:600	Jackson ImmunoResearch
Mouse IgG, Alexa 488 conjugated	Goat	1:600	Molecular Probes
Mouse IgG, Cy3 conjugated	Goat	1:600	Jackson ImmunoResearch
Chicken IgG, Alexa 488 conjugated	Goat	1:600	Jackson ImmunoResearch
Rat IgG, Alexa 488 conjugated	Goat	1:600	Molecular Probes
Rat IgG, Cy3 conjugated	Goat	1:600	Jackson ImmunoResearch
Guinea Pig IgG, Alexa 488 conjugated	Goat	1:600	Molecular Probes
Guinea Pig, Cy5 conjugated	Goat	1:600	Jackson ImmunoResearch
